# *Aspergillus oryzae*-based cell factory for direct kojic acid production from cellulose

**DOI:** 10.1186/1475-2859-13-71

**Published:** 2014-05-18

**Authors:** Ryosuke Yamada, Toshihide Yoshie, Satoshi Wakai, Nanami Asai-Nakashima, Fumiyoshi Okazaki, Chiaki Ogino, Hiromoto Hisada, Hiroko Tsutsumi, Yoji Hata, Akihiko Kondo

**Affiliations:** 1 Organization of Advanced Science and Technology, Kobe University, 1-1 Rokkodaicho, Nada-ku, Kobe, Hyogo 657-8501, Japan; 2 Department of Chemical Science and Engineering, Graduate School of Engineering, Kobe University, 1-1 Rokkodaicho, Nada-ku, Kobe, Hyogo 657-8501, Japan; 3 Research Institute, Gekkeikan Sake Co. Ltd, 101 Shimotoba-koyanagi-cho, Fushimi-ku, Kyoto, Kyoto 612-8385, Japan; 4 Present address: Department of Chemical Engineering, Osaka Prefecture University, 1-1 Gakuencho, Naka-ku, Sakai, Osaka 599-8531, Japan

**Keywords:** *Aspergillus oryzae*, Kojic acid, Cellulose, Cellulase, Starch

## Abstract

**Background:**

Kojic acid (5-Hydroxy-2-(hydroxymethyl)-4-pyrone) is one of the major secondary metabolites in *Aspergillus oryzae*. It is widely used in food, pharmaceuticals, and cosmetics. The production cost, however, is too high for its use in many applications. Thus, an efficient and cost-effective kojic acid production process would be valuable. However, little is known about the complete set of genes for kojic acid production. Currently, kojic acid is produced from glucose. The efficient production of kojic acid using cellulose as an inexpensive substrate would help establish cost-effective kojic acid production.

**Results:**

A kojic acid transcription factor gene over-expressing the *A. oryzae* strain was constructed. Three genes related to kojic acid production in this strain were transcribed in higher amounts than those found in the wild-type strain. This strain produced 26.4 g/L kojic acid from 80 g/L glucose. Furthermore, this strain was transformed with plasmid harboring 3 cellulase genes. The resultant *A. oryzae* strain successfully produced 0.18 g/L of kojic acid in 6 days of fermentation from the phosphoric acid swollen cellulose.

**Conclusions:**

Kojic acid was produced directly from cellulose material using genetically engineered *A. oryzae*. Because *A. oryzae* has efficient protein secretion ability and secondary metabolite productivity, an *A. oryzae*-based cell factory could be a platform for the production of various kinds of bio-based chemicals.

## Background

Kojic acid (5-Hydroxy-2-(hydroxymethyl)-4-pyrone) is widely used in the food industry, pharmaceutical industry, and in cosmetics [1,2]. Furthermore, kojic acid is also used as a building block for biodegradable plastics [3,4]. Plastics have phenolic hydroxyl groups that are derived from kojic acid. Thus, it has some unique properties. Futamura et al. [5] reported that the production cost of kojic acid is about $10/kg. It must be decreased to less than $2/kg before it could be used in many applications. Thus, an efficient and cost-effective kojic acid production process would be desirable.

The utilization of biomass, particularly cellulosic materials, is desirable because it is abundant, inexpensive, renewable, and has favorable environmental properties. However, an efficient and cost-effective method for the degradation of cellulosic materials into glucose would be required before it could be used for the production of bio-based chemicals.

Cellulose has a structure that is very rigid with a high degree of crystallinity. Thus, the degradation of cellulose requires large amounts of various types of cellulase. The degradation of cellulose requires a synergistic reaction of at least three types of cellulase: endoglucanase (EG), cellobiohydrolase (CBH), and β-glucosidase (BGL). Although many studies have reduced the cost of cellulosic material degradation using recombinant bacteria, fungi, and yeast [6], cellulase degradation efficiency has not been sufficiently improved. The filamentous fungus *Trichoderma reesei* degrades cellulose effectively and is known to produce more than several dozen g/L of cellulase in the medium [7], therefore, it is known for its high cellulose degradation ability. *Aspergillus oryzae* is known as a high protein-secreting microorganism and its genetic recombination procedures are well developed. There are many reports concerning the production of a large amount of protein by *A. oryzae*[8]. Thus, *A. oryzae* shows promise as a host strain that could produce a large amount of cellulase and bio-based chemicals from cellulose. There have been reports concerning a single cellulase gene expression by *A. oryzae*, but no studies have focused on the cultivation of the 3 types of cellulase expressing *A. oryzae* on cellulose as a sole carbon source.

Kojic acid is one of the major secondary metabolites in *A. oryzae*. In the 100 years since it was discovered, there has been no elucidation of the entire pathway of kojic acid biosynthesis in *A. oryzae*. Recently, some genes regarding the production of kojic acid in *A. oryzae* were reported [9]. According to the report, 14 genes from AO090113000132 to AO090113000145 including a transcription factor gene (*kojR*; AO090113000137), an enzyme gene (*kojA*, AO090113000136), and a transporter gene (*kojT* AO090113000138) form a cluster. These genes are engaged in kojic acid production. The *kojR* gene encodes a fungal-specific Zn(II)_2_Cy_6_ transcription factor that is located between *kojA* (upstream 743 bp) and *kojT* (downstream 383 bp). The kojR regulates the transcription of *kojA* and *kojT*. A strain with a disrupted transcription factor gene could not produce kojic acid at all [10]. Thus, *kojR* seems to be a key factor in kojic acid production.

The goal of the present study was to construct an *A. oryzae*-based cell factory for direct kojic acid production from cellulose. First, *A. oryzae* over-expressing the transcription factor gene *kojR* was constructed, and kojic acid production from glucose and starch by the resultant strain was carried out. Then, *A. oryzae* co-expressing the transcription factor and 3-types of cellulase genes was constructed. Finally, direct kojic acid production from cellulose by the resultant strain was carried out.

## Methods

### Strains and media

Table 1 summarizes the genetic properties of all strains used. The *A. oryzae* strain RIB40 [11] was used as the wild-type strain. The strain NSPlD1 [12] derived from *A. oryzae* RIB40 was used as a transformation host strain. Potato dextrose agar (PDA; Nissui Pharmaceutical, Tokyo, Japan) media and GPY medium (3% glucose, 0.2% KCl, 0.1% KH_2_PO_4_, 0.05% MgSO_4_ · 7H_2_O, 1% peptone, and 0.5% yeast extract, pH 6.0) were used for the cultivation of *A. oryzae*. Czapek-Dox (CD) medium (2% glucose, 0.3% NaNO_3_(CD-NO_3_), 0.2% KCl, 0.1% KH_2_PO_4_, 0.05% MgSO_4_ · 7H_2_O, and 0.8 M NaCl, 1.5% agar, pH 6.0) with a required supplement (0.0015% methionine, 20 mM uridine, 0.2% uracil) was used for the *niaD*-based plasmid transformation. CD medium was used for *sC*-based plasmid transformation.

**Table 1:** Strains and plasmid used in this study

**Strains and plasmids**	**Relevant features**	**Reference**
*Escherichia coli* strain		
NovaBlue	*endA1 hsdR17(r K12-m K12+) supE44 thi-I gyrA96 relA1 lac recA1/F’*[*proAB + lacIq* ZΔM15::Tn10(Tetr)]	Novagen
*Aspergillus oryzae* strains		
RIB40	Wild type	Machida et al. [11]
NSPlD1	*niaD*^*-*^*sC*^*-*^*adeA*^*-*^*ΔargB::adeA*^*-*^*ΔligD::argB ΔpyrG::adeA*	Maruyama and Kitamoto [12]
NSPlD1/pIS1-kojR	*niaD*^*-*^*::pIS1-kojR [P-sodM::kojR::T-glaB] sC*^*-*^*adeA- ΔargB::adeA- ΔligD::argB ΔpyrG::adeA*	This study
NSPlD1/pIS1-kojR/pIS1sC-CBHI-EGI-BGLI	*niaD*^*-*^*::pIS1-kojR [P-sodM::kojR::T-glaB] sC- ::pIS1sC-CBHI-EGI-BGLI [P-svaA::CBHI::T-svaA, P-hlyA::EGI::T-hlyA, P-sodM::CutL-BGLI::T-glaB] adeA- ΔargB::adeA- ΔligD::argB ΔpyrG::adeA*	This study
Plasmids		
pISI-CBHI	*CBHI* gene from *T. reesei* containing plasmid	This study
pIS1-svaA-CBHI	Vector for expression of *CBHI* from *T. reesei* [*P-svaA::CBHI::T-glaB*]; *niaD* marker	This study
pISI-EG	EGI (*T. reesei*) gene containing plasmid	This study
pIS1-hlyA-EGI	Vector for expression of *EGI* from *T. reesei* [*P-hlyA::EG::T-hlyA*]; *niaD* marker	This study
pIS1-BGLI	Vector for expression *BGLI* from *A. aculeatus* [*P-sodM::CutL-BGL::T-glaB*]; *niaD* marker	This study
pIS1-CBHI-EGI-BGLI	Vector for expression of *CBHI*, *EGI*, and *BGLI* [*P-svaA::CBHI::T-svaA, P-hlyA::EGI::T-hlyA, P-sodM::CutL-BGLI::T-glaB*]; *niaD* marker	This study
pIS1sC-CBHI-EGI-BGLI	Vector for expression *CBHI*, *EGI,* and *BGLI* [*P-svaA::CBHI::T-svaA, P-hlyA::EGI::T-hlyA, P-sodM::CutL-BGLI::T-glaB*]; *sC* marker	This study
pIS1-kojR	Vector for expression of transcription factor (AO090113000137) [*P-sodM::kojR::T-glaB*]; *niaD* marker	This study

GY medium (8% glucose, 0.25% yeast extract, 0.1% K_2_HPO_4_, 0.05% MgSO_4_ · 7H_2_O, pH 6.0), SY medium (8% soluble starch, 0.25% yeast extract, 0.1% K_2_HPO_4_, 0.05% MgSO_4_ · 7H_2_O, pH 6.0), and CY medium (1% phosphoric acid swollen cellulose (PASC), 0.25% yeast extract, 0.1% K_2_HPO_4_, 0.05% MgSO_4_ · 7H_2_O, pH 6.0) was used for kojic acid production. PASC was prepared from Avicel PH-101 (Fluka Chemie GmbH, Buchs, Switzerland) as amorphous cellulose [13]. The transformant and wild-type strains used for kojic acid production with GY medium and SY medium were cultivated in 500 mL Erlenmeyer flasks containing 100 mL medium at 30°C that was shaken at 150 rpm using an orbital shaker with inocula of 1 × 10^5^ spores/mL. The transformant and wild-type strain used for kojic acid fermentation in the CY medium was precultivated in a 200 mL Erlenmeyer flask with baffles containing 100 mL GPY medium at 30°C that was shaken at 150 rpm using an orbital shaker with inocula of 1 × 10^5^ spores/mL. The mycelium in culture medium was separated from the culture medium by filtration using Miracloth (Calbiochem, Darmstadt, Germany) and was washed by distilled water. The washed mycelium was recultivated in 500 mL Erlenmeyer flasks with baffles containing 100 mL CY medium at 30°C that were shaken at 150 rpm using an orbital shaker.

*E. coli* NovaBlue (Novagen, Inc., Madison, WI, USA) was used as the cloning host for recombinant DNA manipulations. The bacterium was grown in Luria–Bertani medium (1% tryptone, 0.5% yeast extract, and 0.5% NaCl) containing 0.1 mg/mL of ampicillin.

### Construction of plasmids

The primers used in the present study are summarized in Table 2. The PCR amplification of DNA fragments was performed using KOD plus neo DNA polymerase (Toyobo, Osaka, Japan).

**Table 2:** Polymerase chain reaction primers used in this study

**Primers**	**Sequence (5′-3′)**	**Template DNA**
ascI-kojR-F	AAAAAGGCGCGCCATGTCGTTGAATACCGACGATTCC	*A. oryzae* genome DNA
notI-FLAG-kojR-R	AAAGCGGCCGCTTACTTGTCATCATCGTCCTTATAGTCTCTATATCTCTGACCACCTGC	
CBHI-F	CCAAACCACCCAAAGGGCGCGCCATGTATCGGAAGTTGGCCGTCATC	synthesized gene
CBHI-R	GAAAGTACATGTCGAGCGGCCGCTTACAGGCACTGAGAGTAGTAAG	
psvaA-ascI-CBHI-F	GGCGCGCCAAAAAATGTATCGGAAGTTGGCCGTCA	pISI-CBHI
tsvaA-notI-CBHI-R	AAATGATGCGGCCGCTTACAGGCACTGAGAGTAGT	
pIS1-psvaA-F	CACAACACTCTCGACCTGCAGCGGTTTACACCGAAGACCGG	*A. oryzae* genome DNA
CBHI-ascI-psvaA-R	ATTTTTTGGCGCGCCCTTGCGAGCAGGGGGATAAT	
CBHI-notI-tsvaA-F	GCGGCCGCATCATTTTCCCGCTTTGATCTGGTCGGTTCCC	*A. oryzae* genome DNA
pIS1-tsvaA-R	GGTACCCGGGGATCCTCTAGATCAAGCATAACTACAACAGGGCAAGGAATACGG	
EGI-F	CCAAACCACCCAAAGGGCGCGCCATGGCGCCCTCAGTTACACTGC	*T. reesei* cDNA
EGI-R	GAAAGTACATGTCGAGCGGCCGCCTAAAGGCATTGCGAGTAGTCTG	
phlyA-ascI-EGI-F	GGCGCGCCAAAAAATGGCGCCCTCAGTTACACTGC	pISI-EG
thlyA-notI-EGI-R	AGAGAGAGCGGCCGCCTAAAGGCATTGCGAGTAGT	
pIS1-phlyA-F	CACAACACTCTCGACCTGCAGTACAGCATGGTCTGGATTCCAATCCACGCAGC	*A. oryzae* genome DNA
EGI-ascI-phlyA-R	ATTTTTTGGCGCGCCGGTGTTGTGGTGTGAAGGGTGATTGATGTGAGACC	
EGI-notI-thlyA-F	GCGGCCGCTCTCTCTCCCCTATACGTGATACCGTA	*A. oryzae* genome DNA
pIS1-thlyA-R	GGTACCCGGGGATCCTCTAGAGATGCAAATTGGAGTTAAAT	
sCutL-F	CCAAACCACCCAAAGGGCGCGCCATGCATCTTGCTATCAAGTCTC	*A. oryzae* genome DNA
sCutL-R	TCACTAGTTCTCTCAACCAGAGCATTGCTGGG	
N28-F	TTGAGAGAACTAGTGATGACAACTTGGTTGGTGGCATG	pISI-GFP (Adachi et al. [14])
N28-R	AGTTCATCCTCGACACGCTTGGCGCTGTTGG	
BGL-F	GTGTCGAGGATGAACTGGCGTTCTCTCCTC	pdU-PGAGBGL (Yamada et al. [15])
BGL-R	GAAAGTACATGTCGAGCGGCCGCTTACTTGTCATCGTCATCCTTG	
pIS1-svaA-CBHI-F	GGCTTTCCCCGTCAAGCTCT	pISI-svaA-CBHI
pIS1-svaA-CBHI-R	GGCGAACGTGGCGAGAAAGG	
phlyA-into-pIS1-svaA-CBHI-F	CTCGCCACGTTCGCCTACAGCATGGTCTGGATTCCAATCCACGCA	pISI-hlyA-EGI
connect-thlyA-and-psodM-R	TATTTAAATATCGATGATGCAAATTGGAGTTAAATATTAACTAAC	
connect-psodM-and-thlyA-F	ATCGATATTTAAATATTATGTACTCCGTACTCGGTTGATTATTAA	pIS1-BGL1
tglaB-into-pIS1-svaA-CBHI-R	TTGACGGGGAAAGCCGGATGTAGTATGTATACTTAGTTTGATTGC	
hlyA-EGI-svaA-CBHI-sodM-BGLI-F	TTTATATCCAAGATCACCTGCAGCGGTTTACACCGAAGACCGGTA	pISI-CBHI-EGI-BGL1
hlyA-EGI-svaA-CBHI-sodM-BGLI-R	TGCCAAGAGAAGCTTGGCGTAATCATGGTCATAGCTGTTT	
sC-F	AAGCTTCTCTTGGCAATAGCTGCCC	pUSC (Yamada et al. [16])
sC-R	GATCTTGGATATAAAAATCCAAATATGGCTCCTCG	
kojR-RT-PCR-F	TGGTGCAATCAGCGAAGGA	wild type and engineered *A. oryzae* cDNA
kojR-RT-PCR-R	AGACTACTCTCCTGCATCATGCC	
kojA-RT-PCR-F	ATCCGAAGGCGAATGGTTT	wild type and engineered *A. oryzae* cDNA
kojA-RT-PCR-R	ATGAACCCAGCGTCGCTATT	
kojT-RT-PCR-F	CATGGTGCCGCATATTTACTTC	wild type and engineered *A. oryzae* cDNA
kojT-RT-PCR-R	AATGGACACAATGGGTTGCC	
ß-Tubulin-RT-PCR-F	GCCAGTGTGGTAACCAAATAGGT	wild type and engineered *A. oryzae* cDNA
ß-Tubulin-RT-PCR-R	TAAACACCGGAGCCGTCAA	

The transcription factor gene (*kojR*, AO090113000137) expression plasmid was constructed as follows. The *kojR* was amplified by PCR using primers ascI-kojR-F and notI-FLAG-kojR-R from *A. oryzae* genome DNA as a template. The amplified fragment was inserted into the AscI/NotI sites of the digested pISI, which contained the *sodM* promoter and *glaB* terminator from *A. oryzae*[17]. The resultant plasmid was named pISI-kojR.

The *CBHI* gene from *Trichoderma* sp. expressing plasmid pIS1-svaA-CBHI was constructed as follows. The DNA fragment encoding the *Trichoderma* sp. CBHI gene (GenBank accession no. X69976) was amplified by PCR using primers CBHI-F and CBHI-R from a synthesized gene (GENEWIZ, South Plainfield, NJ, USA). The fragment was inserted into the AscI/NotI site of the plasmid pISI using an In-Fusion HD Cloning Kit (Takara Bio Inc., Shiga, Japan). The resultant plasmid was named pISI-CBHI. Then, the *CBHI* gene was amplified by PCR using primers psvaA-ascI-CBHI-F and tsvaA-notI-CBHI-R with pISI-CBHI as a template. The *svaA* promoter [17] and *svaA* terminator were amplified by PCR using primers pIS1-psvaA-F and CBHI-ascI-psvaA-R, and CBHI-notI-tsvaA-F and pIS1-tsvaA-R, respectively, with *A. oryzae* genome DNA as a template. These PCR-amplified DNA fragments were simultaneously inserted into the XbaI and PstI sites of pIS1 using an In-Fusion PCR Cloning kit. The resultant plasmid was named pISI-svaA-CBHI.

An *EGI* gene from the *T. reesei-*expressing plasmid pIS1-hlyA-EGI was constructed. The EGI-expressing plasmid was constructed as follows. The DNA fragment encoding the *T. reesei EGI* gene was amplified by PCR using the primers EGI-F and EGI-R with *T. reesei* cDNA as a template. The fragment was inserted into the AscI/NotI site of the plasmid pISI using an In-Fusion HD Cloning Kit. The resultant plasmid was named pISI-EG. The *T. reesei EGI* gene was then amplified by PCR using the primers phlyA-ascI-EGI-F and thlyA-notI-EGI-R with pISI-EG as a template. The hlyA promoter [18] and hlyA terminator were amplified by PCR using primers pIS1-phlyA-F and EGI-ascI-phlyA-R, and EGI-notI-thlyA-F and pIS1-thlyA-R, respectively, using the *A. oryzae* genome DNA as a template. These PCR-amplified DNA fragments were simultaneously inserted into the XbaI and PstI sites of pIS1 using an In-Fusion PCR Cloning kit. The resultant plasmid was named pISI-hlyA-EGI.

The BGL1 gene from *A. aculeatus* expressing plasmid pIS1-BGL1 was constructed as follows. The DNA fragment encoding the secretion signal from *A. oryzae* cutinase, the 28 amino acids from the N-terminal region of *Rhizopus oryzae* lipase, and the *A. aculeatus* BGL gene were amplified by PCR using the primers sCutL-F and sCutL-R, N28-F and N28-R, and BGL-F and BGL-R, respectively, from the genome DNA of *A. oryzae*, pISI-GFP [14], and pδU-PGAGBGL [15], respectively. These fragments were simultaneously inserted into the AscI/NotI site of the plasmid pISI using an In-Fusion HD Cloning Kit. The resultant plasmid was named pISI-BGL.

The pIS1-CBHI-EGI-BGL1 plasmid was constructed as follows. The pISI-svaA-CBHI was linearized by PCR using the primers pIS1-svaA-CBHI-F and pIS1-svaA-CBHI-R. The EGI- and BGL1-expressing cassettes were amplified by PCR using primers phlyA-into-pIS1-svaA-CBHI-F and connect-thlyA-and-psodM-R, and connect-psodM-and-thlyA-F and tglaB-into-pIS1-svaA-CBHI-R, respectively, from pISI-hlyA-EGI and pIS1-BGL1, respectively. These fragments were fused using an In-Fusion PCR Cloning kit. The resultant plasmid was named pISI-CBHI-EGI-BGL1.

The pIS1sC-CBHI-EGI-BGL1 plasmid was constructed as follows. The CBHI-, EGI-, and BGL1-expressing cassettes were amplified by PCR using primer (hlyA-EGI-svaA-CBHI-sodM-BGL1-F and hlyA-EGI-svaA-CBHI-sodM-BGL1-R). The *sC* gene was amplified by PCR using primer (sC-F and sC-R) from a pUSC plasmid [16]. These amplified fragments were fused using an In-Fusion PCR Cloning System. The resultant plasmid was named pISIsC-CBHI-EGI-BGL1.

### Transformation of *A. oryzae*

The transformation of *A. oryzae* was carried out according to the previously described method with minor modifications [19].

The niaD-based pIS1-kojR plasmids were introduced into the NSPlD1 strain. The resultant strain was named NSPlD1/pIS1-kojR. The *sC*-based plasmid pIS1sC-CBHI-EGI-BGL1 was introduced into the NSPlD1/pIS1-kojR strain. The resultant strain was named NSPlD1/pIS1-kojR/pIS1sC-CBHI-EGI-BGL1.

### RNA extraction and reverse transcription

The RNA was isolated from fungus bodies cultivated in GY medium for 6 days at 30°C using NucleoSpin RNA II (Takara Bio Inc.) according to the manufacturer’s protocol. The cDNA synthesis was carried out using a ReverTra Ace qPCR RT Kit (Toyobo) according to the manufacturer’s protocol.

### Real-time PCR analysis

The transcription level of Kojic-acid-producing genes (*kojR, kojA, kojT*) was quantified by real-time PCR. Quantitative real-time PCR was performed using an Mx3005P Real-Time QPCR System (Agilent Technologies, Santa Clara, CA, USA) with a Thunderbird SYBR qPCR Mix (Toyobo). The normalized transcription level was calculated using the standard curve method with β-tubulin as the house-keeping gene [20]. All primers used for real-time PCR analysis are summarized in Table 2.

### Enzyme assay

A wild-type strain and 3-types of cellulase gene-expressing strains were cultivated in GY medium for 6 days at 30°C, and the culture supernatant was used for the determination of PASC degradation activity. PASC degradation activity was measured in 25 mM sodium acetate buffer (pH 5.0) at 30°C with 5 g/L of PASC. After hydrolysis, the supernatant was separated by centrifugation for 5 min at 10,000 × *g* and 4°C, and the produced glucose concentration was measured. One unit of PASC degradation activity was defined as the amount of enzyme producing 1 μmol/min glucose at 30°C, pH 5.0.

### Analytical methods

The kojic acid concentration was determined using a colorimetric method described previously [21]. The total sugar concentration was determined by a colorimetric phenol-sulfuric acid method described previously [22]. The glucose concentration was determined using a Wako Glucose CII-Test kit (Wako Pure Chemical Industries, Osaka, Japan).

## Results

### Kojic acid production from glucose using the engineered *A. oryzae*

**Figure 1:**
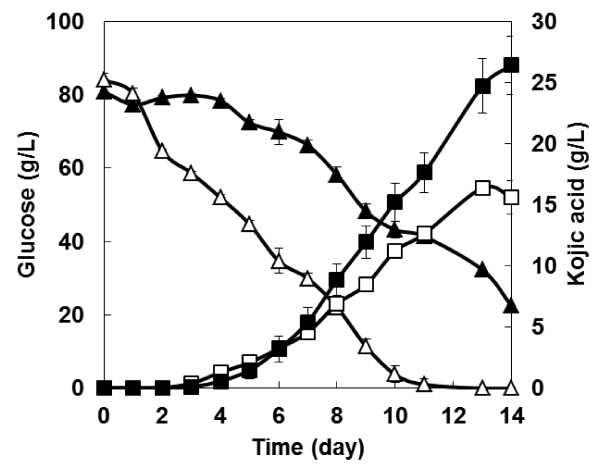
**Time course of kojic acid production from glucose.** Triangles, glucose; squares, kojic acid; open symbols, wild-type strain; and, closed symbols, transcription factor over-expressing strain NSPlD1/pIS1-kojR. The data represent the averages from three independent experiments (error bars represent SE).

To confirm the effect of the over-expression of the transcription factor for kojic acid production, the kojic acid productivity from the transcription factor over-expressing strain NSPlD1/pIS1-kojR from glucose was evaluated. As shown in Figure 1 the wild-type strain produced 16.4 g/L of kojic acid after 14 days of fermentation. By contrast, the transcription factor over-expressing strain NSPlD1/pIS1-kojR produced 26.4 g/L of kojic acid after 14 days of fermentation and it was 1.6-fold higher than that of the wild-type strain.

### Quantification of the transcription level of kojic-acid-producing genes by real-time PCR

**Figure 2:**
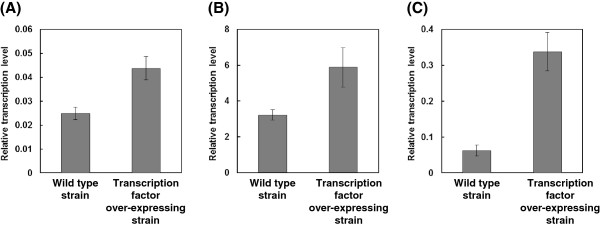
**Transcription levels of kojic acid producing genes. (A)** Transcription factor gene, **(B)** enzyme gene, **(C)** transporter gene. The data represent the averages from three independent experiments (error bars represent SE).

To confirm the effect of the over-expression of transcription factor at the level of the kojic-acid-producing genes, the transcription level of the transcription factor, the kojic-acid-producing enzyme, and the transporter gene were evaluated by real-time PCR. As shown in Figure 2, the transcription factor of the over-expressing strain NSPlD1/pIS1-kojR showed a 1.8, 1.8, and 5.4-fold increases in the transcription level of transcription factor, enzyme, and transporter gene, respectively, compared with the wild-type strain.

### Kojic acid production from soluble starch by recombinant *A. oryzae*

**Figure 3:**
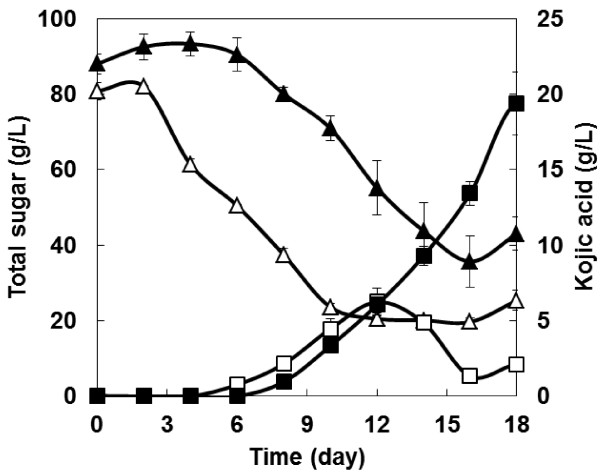
**Time course of kojic acid production from starch.** Triangles, total sugar; squares, kojic acid; open symbols, wild-type strain; and, closed symbols, transcription factor over-expressing strain NSPlD1/pIS1-kojR. The data represent the averages from three independent experiments (error bars represent SE).

Since *A. oryzae* can assimilate starch as a sole carbon source intrinsically, direct kojic acid production from starch using the transcription factor over-expressing strain NSPlD1/pIS1-kojR was carried out. As shown in Figure 3, the wild-type strain produced 6.2 g/L of kojic acid after 18 days of fermentation. In contrast, the transcription factor over-expressing strain produced 19.4 g/L of kojic acid after 18 days of fermentation, i.e. 3.1-fold gain.

### Evaluation of cellulase activity

**Figure 4:**
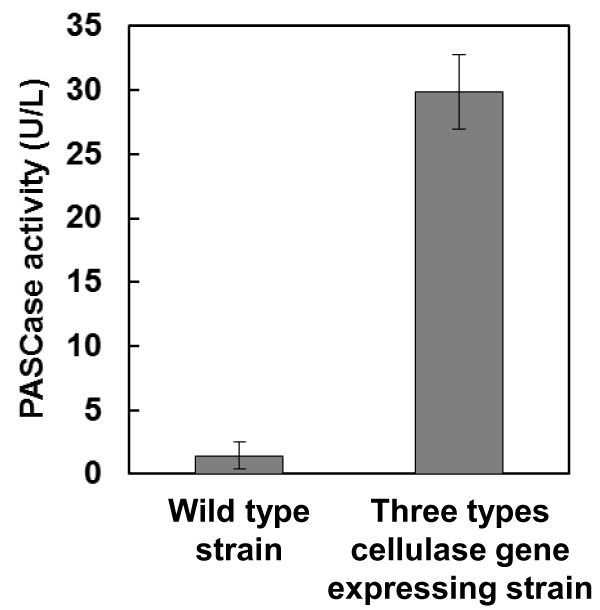
**PASCase activity of 3-types of cellulase gene-expressing strains.** The data represent the averages from three independent experiments (error bars represent SE).

To confirm the cellulase expression of engineered *A. oryzae*, the PASC degradation activity of 3-types of cellulase were evaluated. As shown in Figure 4, the 3-types of cellulase gene-expressing strains NSPlD1/pIS1-kojR/pIS1sC-CBHI-EGI-BGLI showed 29.9 U/L after 6 days of cultivation. In contrast, the wild-type strain showed significantly lower PASCase activity (1.4 U/L).

### Direct kojic acid production from PASC

**Figure 5:**
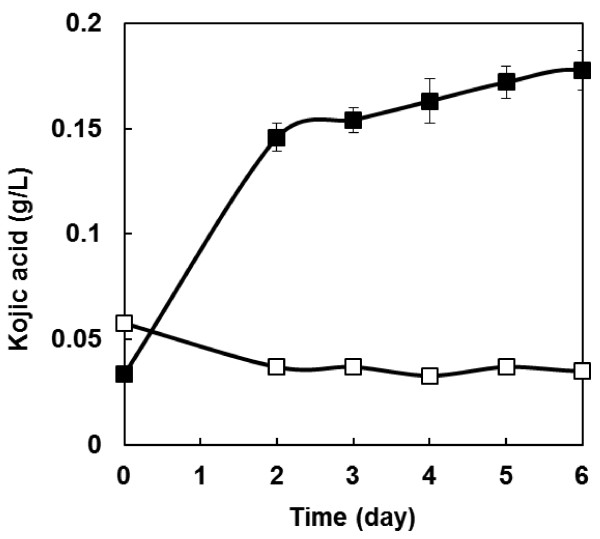
**Time course of kojic acid production from PASC.** Open symbols, wild-type strain; closed symbols, transcription factor and 3-types of cellulase gene over-expressing strains NSPlD1/pIS1-kojR/pIS1sC-CBHI-EGI-BGLI. The data represent the averages from three independent experiments (error bars represent SE).

To confirm direct kojic acid productivity from cellulose, kojic acid production from PASC was carried out. As shown in Figure 5, there was 0.18 g/L of kojic acid produced after 6 days of fermentation when using a transcription factor gene and the 3-types of cellulase genes co-expressing strain NSPlD1/pIS1-kojR/pIS1sC-CBHI-EGI-BGLI. In contrast, the wild-type strain did not produce a detectable amount of kojic acid.

## Discussion

In the present study, a transcription factor gene over-expressing *A. oryzae* NSPlD1/pIS1-kojR was developed by gene recombination strategy. Furthermore, the transcription factor gene and 3-types of cellulase genes co-expressing *A. oryzae* NSPlD1/pIS1-kojR/pIS1sC-CBHI-EGI-BGLI was constructed and the strain successfully produced kojic acid directly from PASC.

The transcription factor gene *kojR*, which is responsible for kojic acid production, was over-expressed in this study, and as a result, kojic acid productivity was improved. As shown in Figure 2, the transcription levels of both the kojic acid-producing enzyme *kojA* and the exporting transporter *kojT* were enhanced. This was because the kojic acid productivity was improved. Terabayashi et al. [9] reported that the *kojR*, *kojA*, and *kojT* genes would encode the transcription factor, the kojic acid-producing enzyme, and the transporter, respectively. In the present study, the transcription level of the enzyme and the transporter were enhanced by an over-expression of the transcription factor, which correlates well with that report. Although there are reports of improved kojic acid productivity through fermentation engineering strategies [1,5,23], to our knowledge, this study is the first to report an improvement in kojic acid productivity via genetic engineering. Kojic acid productivity could be improved even more by combining fermentation engineering and genetic engineering in future work.

Direct kojic acid production from cellulose was successfully demonstrated by *A. oryzae* expressing 3-types of cellulase genes. However, the yield of kojic acid remained low. This was due to the low levels of cellulase production. In previous reports, some microorganisms such as *Saccharomyces cerevisiae*, *Kluyveromyces marxianus*, *Bacillus subtilis*, and *E. coli* were used for bio-based chemical production from cellulose by conferring cellulolytic activity [6]. Although most reports did not indicate the amount of cellulase production, Zhang et al. [24] reported that about 14 mg/L of endoglucanase was produced by recombinant *Bacillus subtilis*. In any case, because *T. reesei* produces several dozen g/L of cellulase [7], the amount of cellulase expression from cellulolytic activity conferred microorganisms has not yet reached the level of *T. reesei*. Although *A. oryzae* has an extremely high protein secreting ability, cellulase expression of *T. reesei* is still higher than that of *A. oryzae*. Because the maximum protein production is approximately 20 g/L in *Aspergillus* organisms [25], the cellulase productivity could be improved with genetic engineering.

Three types of cellulase genes and specific transcription factors were successfully over-expressed simultaneously in recombinant *A. oryzae*. To this point, only a few reports have focused on the co-expression of more than 3-kinds of genes in *A. oryzae*. This is because the selection marker for gene recombination is limited in *A. oryzae* compared with other microorganisms such as yeast *S. cerevisiae*[26]. In the present study, a 3-cassette plasmid with different promoters was constructed and the simultaneous overexpression of 4 genes was successful. Thus, the simultaneous multi gene expression technique, which is an important technique for the construction of a cell factory in *A. oryzae*, has been well established. By using this technique, an *A. oryzae*-based cell factory could be a promising strategy for producing various bio-based chemicals.

## Conclusions

The productivity of kojic acid from *A. oryzae* was made more efficient by the over-expression of specific transcription factors. To accomplish this, recombinant *A. oryzae* expressing 3 types of cellulase genes was constructed. Direct kojic acid production from PASC by genetically engineered *A. oryzae* was successfully demonstrated. Although this research was conducted as the proof of a concept, the results showed that an *A. oryzae*-based cell factory could be established. Because, *A. oryzae* has efficient protein secretion ability and secondary metabolite productivity, an *A. oryzae*-based cell factory could be a promising platform for producing various kinds of bio-based chemicals.

## Competing interests

The authors declare that they have no competing interests.

## Authors’ contributions

TY designed and performed the experiments. NAN performed the experiments. TY and RY wrote the paper. SW, FO, CO, HH, HT, YH and AK commented and supervised on the manuscript. All the authors approved the final manuscript.

## References

[B1] Rosfarizan M Ariff AB Hassan MA Karim MI Kojic acid production by *Aspergillus flavus* using gelatinized and hydrolyzed sago starch as carbon sources Folia Microbiol (Praha) 1998 43 459 464 10.1007/BF02820791 9867479

[B2] Wan HM Chen CC Chang TS Giridhar RN Wu WT Combining induced mutation and protoplasting for strain improvement of *Aspergillus oryzae* for kojic acid production Biotechnol Lett 2004 26 1163 1166 15266124 10.1023/B:BILE.0000035490.49252.38

[B3] Tomita I Mitsuhashi K Endo T Synthesis and radical polymerization of styrene derivative bearing kojic acid moieties J Polym Sci A Polym Chem 1996 34 271 276 10.1002/(SICI)1099-0518(19960130)34:2<271::AID-POLA13>3.0.CO;2-N

[B4] Ochiai B Kamiya M Endo T Synthesis and Fe(III)-complexation ability of polyurethane bearing kojic acid skeleton in the main chain prepared by polyaddition of aliphatic hydroxyl groups without protection of phenolic hydroxyl groups J Polym Sci A Polym Chem 2012 50 3493 3498 10.1002/pola.26161

[B5] Futamura T Ishihara H Tamura T Yasutake T Huang G Kojima M Okabe M Kojic acid production in an airlift bioreactor using partially hydrolyzed raw corn starch J Biosci Bioeng 2001 92 360 365 16233111 10.1263/jbb.92.360

[B6] Yamada R Hasunuma T Kondo A Endowing non-cellulolytic microorganisms with cellulolytic activity aiming for consolidated bioprocessing Biotechnol Adv 2013 31 754 763 10.1016/j.biotechadv.2013.02.007 23473971

[B7] Peterson R Nevalainen H Trichoderma reesei RUT-C30–thirty years of strain improvement Microbiology 2012 158 58 68 10.1099/mic.0.054031-0 21998163

[B8] Fleissner A Dersch P Expression and export: recombinant protein production systems for *Aspergillus* Appl Microbiol Biotechnol 2010 87 1255 1270 10.1007/s00253-010-2672-6 20532762

[B9] Terabayashi Y Sano M Yamane N Marui J Tamano K Sagara J Dohmoto M Oda K Ohshima E Tachibana K Higa Y Ohashi S Koike H Machida M Identification and characterization of genes responsible for biosynthesis of kojic acid, an industrially important compound from *Aspergillus oryzae* Fungal Genet Biol 2010 47 953 961 10.1016/j.fgb.2010.08.014 20849972

[B10] Marui J Yamane N Ohashi-Kunihiro S Ando T Terabayashi Y Sano M Ohashi S Ohshima E Tachibana K Higa Y Nishimura M Koike H Machida M Kojic acid biosynthesis in *Aspergillus oryzae* is regulated by a Zn(II)(2)Cys(6) transcriptional activator and induced by kojic acid at the transcriptional level J Biosci Bioeng 2011 112 40 43 10.1016/j.jbiosc.2011.03.010 21514215

[B11] Machida M Asai K Sano M Tanaka T Kumagai T Terai G Kusumoto K Arima T Akita O Kashiwagi Y Abe K Gomi K Horiuchi H Kitamoto K Kobayashi T Takeuchi M Denning DW Galagan JE Nierman WC Yu J Archer DB Bennett JW Bhatnagar D Cleveland TE Fedorova ND Gotoh O Horikawa H Hosoyama A Ichinomiya M Igarashi R Genome sequencing and analysis of *Aspergillus oryzae* Nature 2005 438 1157 1161 10.1038/nature04300 16372010

[B12] Maruyama J Kitamoto K Multiple gene disruptions by marker recycling with highly efficient gene-targeting background (ΔligD) in *Aspergillus oryzae* Biotechnol Lett 2008 30 1811 1817 10.1007/s10529-008-9763-9 18574559

[B13] Den Haan R Rose SH Lynd LR van Zyl WH Hydrolysis and fermentation of amorphous cellulose by recombinant *Saccharomyces cerevisiae* Metab Eng 2007 9 87 94 10.1016/j.ymben.2006.08.005 17112757

[B14] Adachi T Ito J Kawata K Kaya M Ishida H Sahara H Hata Y Ogino C Fukuda H Kondo A Construction of an *Aspergillus oryzae* cell-surface display system using a putative GPI-anchored protein Appl Microbiol Biotechnol 2008 81 711 719 10.1007/s00253-008-1687-8 18813924

[B15] Yamada R Taniguchi N Tanaka T Ogino C Fukuda H Kondo A Cocktail delta-integration: a novel method to construct cellulolytic enzyme expression ratio-optimized yeast strains Microb Cell Fact 2010 9 32 10.1186/1475-2859-9-32 20465850 PMC2876996

[B16] Yamada O Lee BR Gomi K Transformation system for *Aspergillus oryzae* with double auxotrophic mutations, *niaD* and *sC* Biosci Biotechnol Biochem 1997 61 1367 1369 10.1271/bbb.61.1367

[B17] Ishida H Hata Y Kawato A Abe Y Kashiwagi Y Isolation of a novel promoter for efficient protein production in *Aspergillus oryzae* Biosci Biotechnol Biochem 2004 68 1849 1857 10.1271/bbb.68.1849 15388959

[B18] Bando H Hisada H Ishida H Hata Y Katakura Y Kondo A Isolation of a novel promoter for efficient protein expression by *Aspergillus oryzae* in solid-state culture Appl Microbiol Biotechnol 2011 92 561 569 10.1007/s00253-011-3446-5 21732241

[B19] Kitamoto K Molecular biology of the Koji molds Adv Appl Microbiol 2002 51 129 153 12236056 10.1016/s0065-2164(02)51004-2

[B20] McKelvey SM Murphy RA Analysis of wide-domain transcriptional regulation in solid-state cultures of *Aspergillus oryzae* J Ind Microbiol Biotechnol 2010 37 455 469 10.1007/s10295-010-0691-z 20145973

[B21] Bentley R Preparation and analysis of kojic acid Methods Enzymol 1957 3 238 241

[B22] Dubois M Gilles KA Hamilton JK Rebers PA Smith F Colorimetric method for determination of sugars and related substances Anal Chem 1956 28 350 356 10.1021/ac60111a017

[B23] Wan HM Chen CC Giridhar R Chang TS Wu WT Repeated-batch production of kojic acid in a cell-retention fermenter using *Aspergillus oryzae* M3B9 J Ind Microbiol Biotechnol 2005 32 227 233 10.1007/s10295-005-0230-5 15895266

[B24] Zhang XZ Sathitsuksanoh N Zhu Z Percival Zhang YH One-step production of lactate from cellulose as the sole carbon source without any other organic nutrient by recombinant cellulolytic Bacillus subtilis Metab Eng 2011 13 364 372 10.1016/j.ymben.2011.04.003 21549854

[B25] Su X Schmitz G Zhang M Mackie RI Cann IK Heterologous gene expression in filamentous fungi Adv Appl Microbiol 2012 81 1 61 22958526 10.1016/B978-0-12-394382-8.00001-0

[B26] Ruiz-Díez B Strategies for the transformation of filamentous fungi J Appl Microbiol 2002 92 189 195 10.1046/j.1365-2672.2002.01516.x 11849345

